# Introducing JMIR Bioinformatics and Biotechnology: A Platform for Interdisciplinary Collaboration and Cutting-Edge Research

**DOI:** 10.2196/48631

**Published:** 2023-06-12

**Authors:** Ece Dilber Gamsiz Uzun

**Affiliations:** 1 Department of Pathology and Laboratory Medicine Rhode Island Hospital Providence, RI United States; 2 Department of Pathology and Laboratory Medicine Brown University Providence, RI United States

**Keywords:** bioinformatics, biotechnology, artificial intelligence, genomic, informatics, interdisciplinary research

## Abstract

*JMIR Bioinformatics and Biotechnology* supports interdisciplinary research and welcomes contributions that push the boundaries of bioinformatics, genomics, artificial intelligence, and pathology informatics.

## Introduction

Bioinformatics is a rapidly evolving field that has transformed the way we study and understand biological systems. With the advent of large-scale genomic data and advances in computational tools and algorithms, we are now able to discover hidden patterns in biological data. One of the key areas where bioinformatics is making a significant impact is in the detection and interpretation of genomic variations. Genomic variations can have important implications for disease susceptibility, drug response, and other biological processes. With the help of advanced algorithms and tools, researchers can now detect genomic variations with high accuracy and precision. This has opened new avenues for drug discovery and precision medicine [1].

The rapid advances in artificial intelligence (AI) applications in the fields of genomics and pathology informatics have led the development of AI-based models for disease risk prediction and drug discovery. AI algorithms can be trained to analyze large-scale genomic data and identify hidden patterns. This has led to significant improvements in disease diagnosis, prognosis, and treatment. AI-based tools can now identify genetic markers that are associated with specific diseases, such as cancer, and help clinicians select the most effective treatment options [2-4].

Network biology is another area where bioinformatics is making significant advances. By analyzing large-scale genomic data sets, researchers can identify key pathways, protein-protein interactions, and networks that are involved in disease pathogenesis. This information can aid in the development of new drugs and therapies [5,6]. Genomic data visualization has led to new and innovative ways for researchers to gain insights into the structure and function of biological systems. This has important implications for understanding disease mechanisms, as well as for developing new diagnostic and therapeutic tools [7]. *JMIR Bioinformatics and Biotechnology* will support the development of new bioinformatics analysis tools, novel algorithms, advanced AI-based predictive models, and network biology studies. We would like to foster not only basic science and algorithm development but also translational research studies in the focus areas described above. We will also explore the use of new technologies for drug discovery and therapy development in cancer and other complex disorders.

## The Scope of JMIR Bioinformatics and Biotechnology

*JMIR Bioinformatics and Biotechnology* aims to publish cutting-edge research in the fields of bioinformatics, genomics, and pathology informatics. The scope of the journal includes the development and application of genomic variation detection algorithms and tools including single-cell sequencing and spatial transcriptomics; AI-based predictive models; pathology informatics, including image analysis; mathematical modeling in biological systems, including drug delivery and discovery; genomic data visualization; network biology; and cancer genomic data analysis. *JMIR Bioinformatics and Biotechnology* will be a platform for interdisciplinary collaborations between bioinformaticians, biologists, computer scientists, mathematicians, and clinicians to address the challenges of integrating large-scale genomic data with clinical and pathological information. The journal welcomes original research articles, review articles, and perspectives, as well as submissions on methodological advances and computational tools in these areas.

Although we welcome translational research studies, *JMIR Bioinformatics and Biotechnology* will not consider manuscripts describing medical informatics–related projects without a focus on bioinformatics or genomics. We aim to focus on the bioinformatics applications within the medical informatics field. We welcome you to *JMIR Bioinformatics and Biotechnology* and hope that you will consider contributing to the fast-moving bioinformatics field!

## Abbreviations

AI: artificial intelligence
